# Data on four apoptosis-related genes in the colonial tunicate *Botryllus schlosseri*

**DOI:** 10.1016/j.dib.2016.05.017

**Published:** 2016-05-20

**Authors:** Nicola Franchi, Francesca Ballin, Lucia Manni, Filippo Schiavon, Loriano Ballarin

**Affiliations:** Department of Biology, University of Padova, Italy

**Keywords:** Sequence databases, Alignments, BLAST analysis, *Botryllus schlosseri*, Apoptosis-related transcripts, Bax, AIF1, PARP1, IAP7

## Abstract

The data described are related to the article entitled “Recurrent phagocytosis-induced apoptosis in the cyclical generation change of the compound ascidian *Botryllus schlosseri*” (Franchi et al., 2016) [1]. Four apoptosis-related genes, showing high similarity with mammalian Bax (a member of the Bcl-2 protein family), AIF1 (apoptosis-inducing factor-1), PARP1 (poly ADP ribose polymerase-1) and IAP7 (inhibitor of apoptosis-7) were identified from the analysis of the trascriptome of *B. schlosseri*. They were named BsBax, BsAIF1, BsPARP1 and BsIAP7. Here, their deduced amino acid sequence were compared with known sequences of orthologous genes from other deuterostome species together with a study of their identity/similarity.

**Specifications Table**

**Table:** 

Subject area	*Biology*
More specific subject area	*Developmental Biology*
Type of data	*Tables, figures*
How data was acquired	*Bioinformatic analysis, RACE*
Data format	*Raw and analysed data*
Experimental factors	*The partial transcripts present in the transcriptome, identified by BLAST analysis, were elongated through 5’and 3’ RACE according to the 2nd generation of 5’/3’ RACE kit*
Experimental features	*Analysis with BLAST, LALIGN, SMART, Clustal Omega*
Data source location	*Padova, Italy*
Data accessibility	*Data are available in this article and at GenBank via accession numbers GenBank: KU948200 for BsBAX, GenBank: KU948201 for BsPARP1, GenBank: KU948202 for BsAIF1, GenBank: KU948203 for BsIAP7.*

**Value of the data**

• The data provide the full-length sequences of four apoptosis-related transcripts from the colonial ascidian *B. schlosseri* useful to study the phylogeny trees of the corresponding proteins in chordates.
• From the data, the protein primary structures can be deduced and, from that, three-dimensional models can be obtained, useful to compare the domain organization of the corresponding chordate proteins.
• Expression studies, exploiting the present data, can contribute to elucidate the dynamics of the cyclical apoptosis, which characterizes the colonial blastogenetic cycle of the ascidian *B. schlosseri*.

## 1 Data

The data reported include supporting information to the phylogenetic analyses of Franchi et al. [1]. They consist of transcript sequences, sequence alignments and comparisons of four apoptosis-related genes identified in the recently-obtained transcriptome of *B. schlosseri* [2]. The sequences show high similarity with mammalian transcripts for Bax, AIF1, PARP1 and IAP7 and were named BsBax, BsAIF1, BsPARP1 and BsIAP7, respectively. The expression of these genes was studied further in the above-reported paper [1].

## 2 Experimental design, materials and methods

Amplification and cloning of transcripts for BsBax, BsAIF1, BsPARP1 and BsIAP7 was achieved with specific primers designed on sequences found in our collection of transcriptomes [2]. In order to verify and complete the full length cDNA, PCR reactions were carried out with a denaturing step at 94 °C for 2 min, 40 cycles of 30 s at 94 °C, 40 s at 60 °C and 90 s at 72 °C, and a final extension at 72 °C for 10 min. Amplicons were separated using 1.5% agarose gel, purified, cloned and sequenced. The partial transcripts were elongated through 5′ and 3′ RACE according to the 2nd generation of 5′/3′ RACE kit (Roche). Supplementary Table 1 reports the specific primers used for amplicons production and their elongation through 5′- and 3′-RACE and for the *in situ* hybridisation experiments reported in [1].

**Fig. 1:**
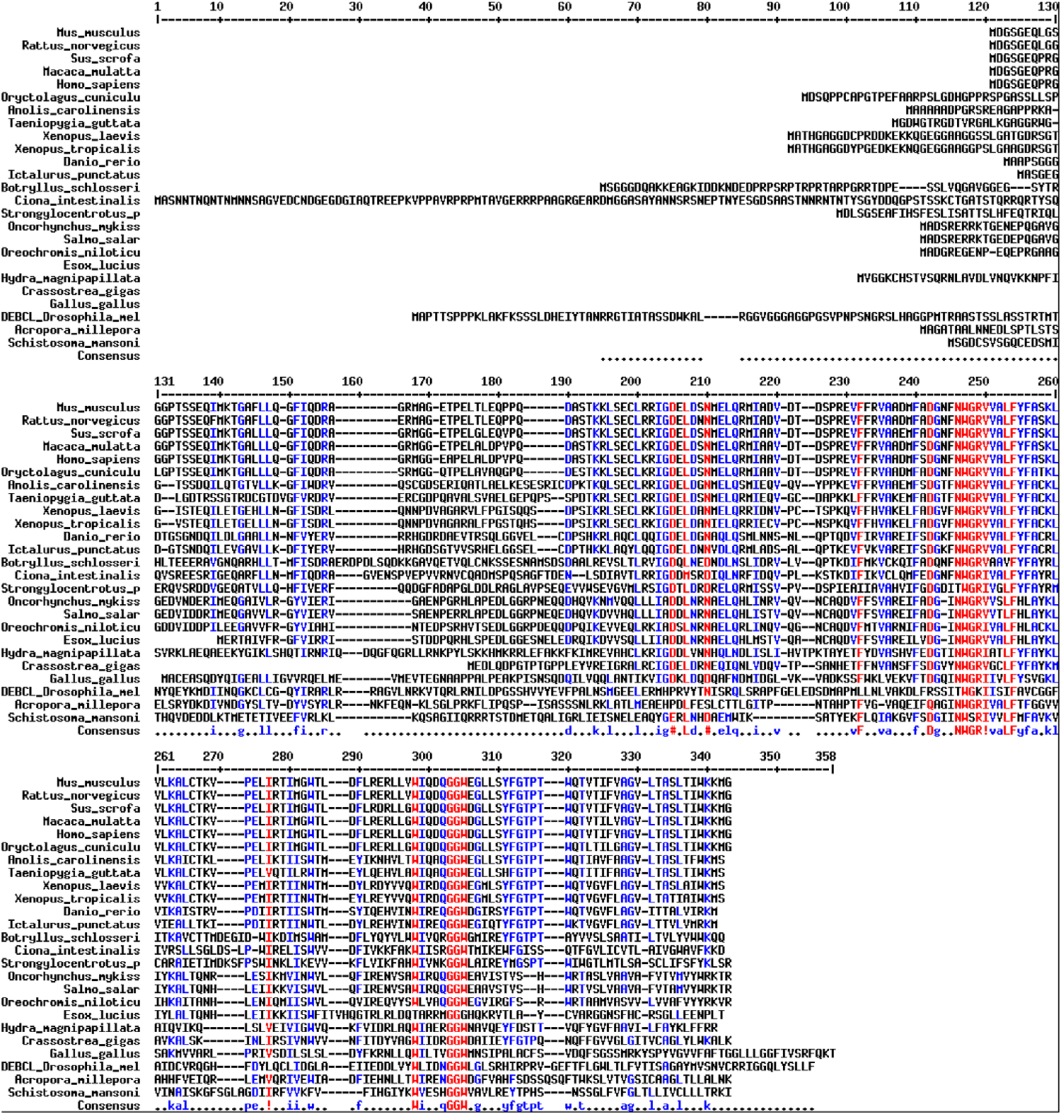
Alignments of the deduced amino acid sequence of BsBax with known orthologous sequences from both vertebrate and invertebrates.

**Fig. 2:**
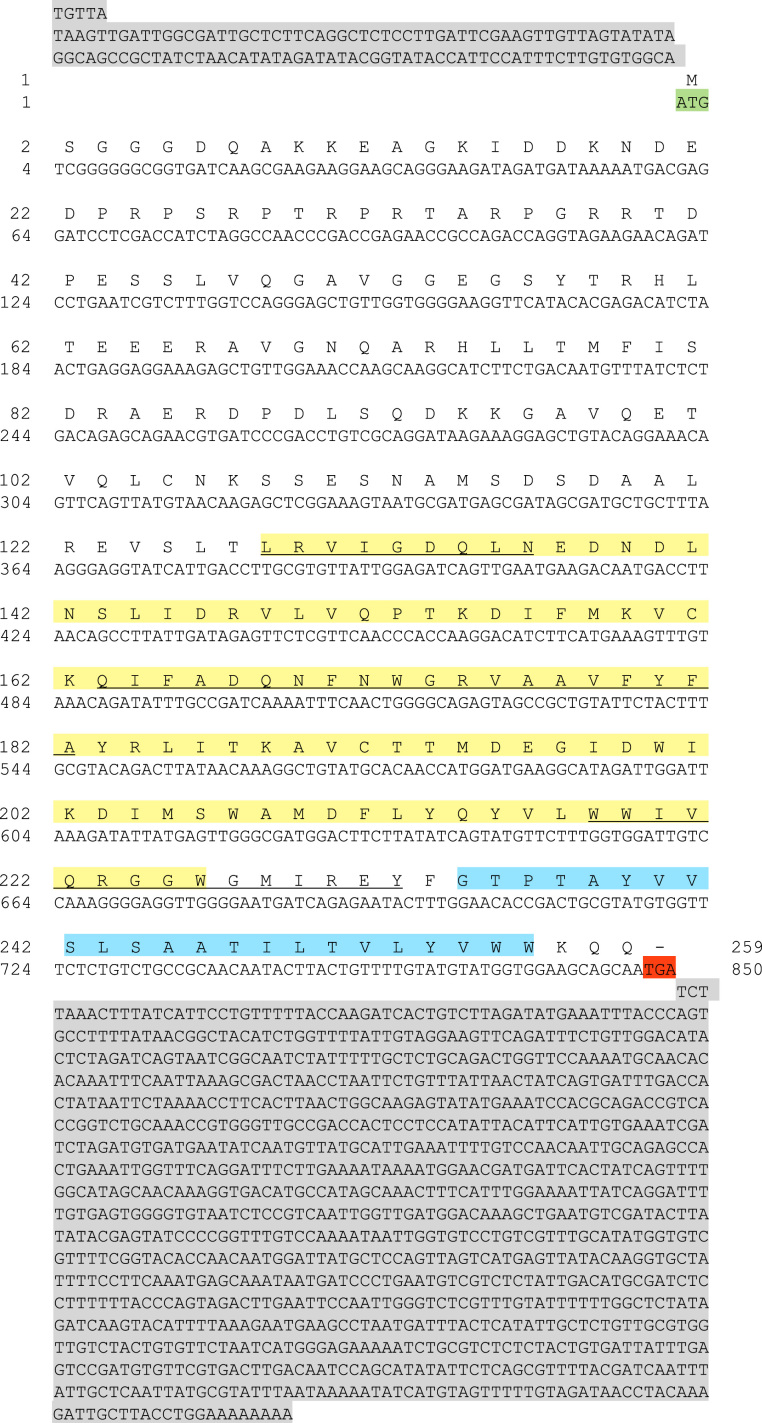
cDNA and deduced amino acid sequence of BsBax. START and STOP codon are in green and red, respectively; 5′ and 3′ UTR are in grey. In yellow, the Bcl-2 domain and, within this, BH1, BH2 and BH3 domains are underlined. Light blue: transmembrane domain.

**Fig. 3:**
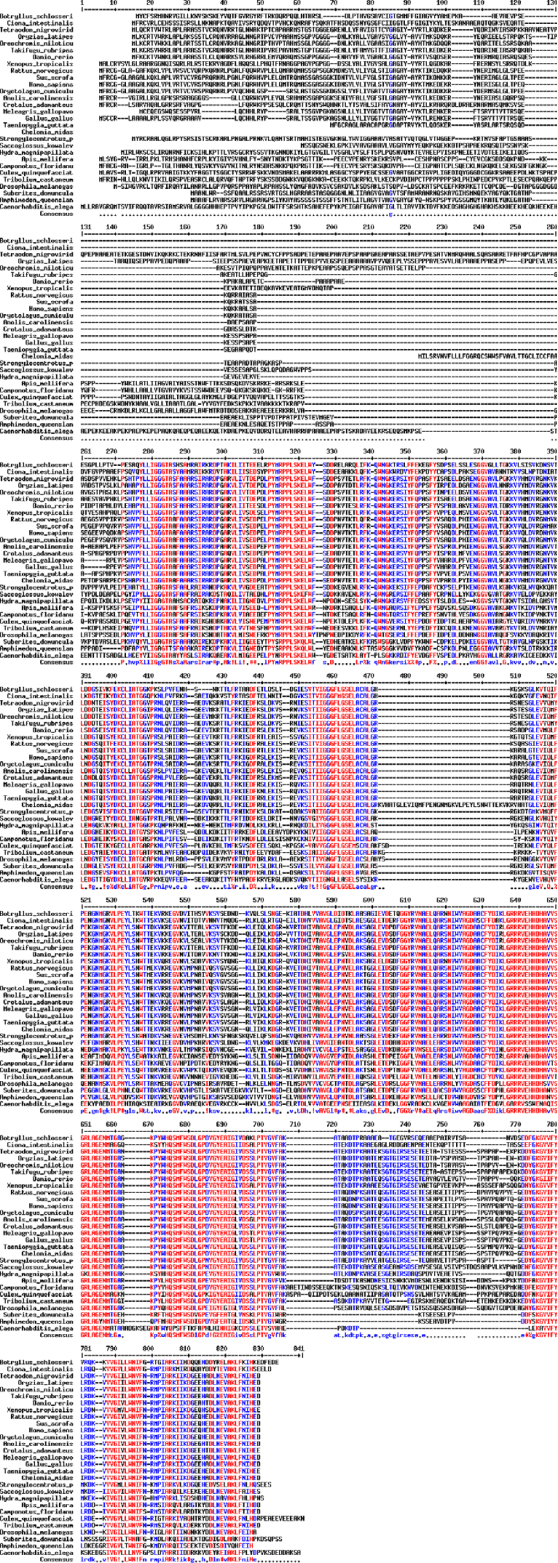
Alignments of the deduced amino acid sequence of BsAIF1 with known orthologous sequences from both vertebrate and invertebrates.

**Fig. 4:**
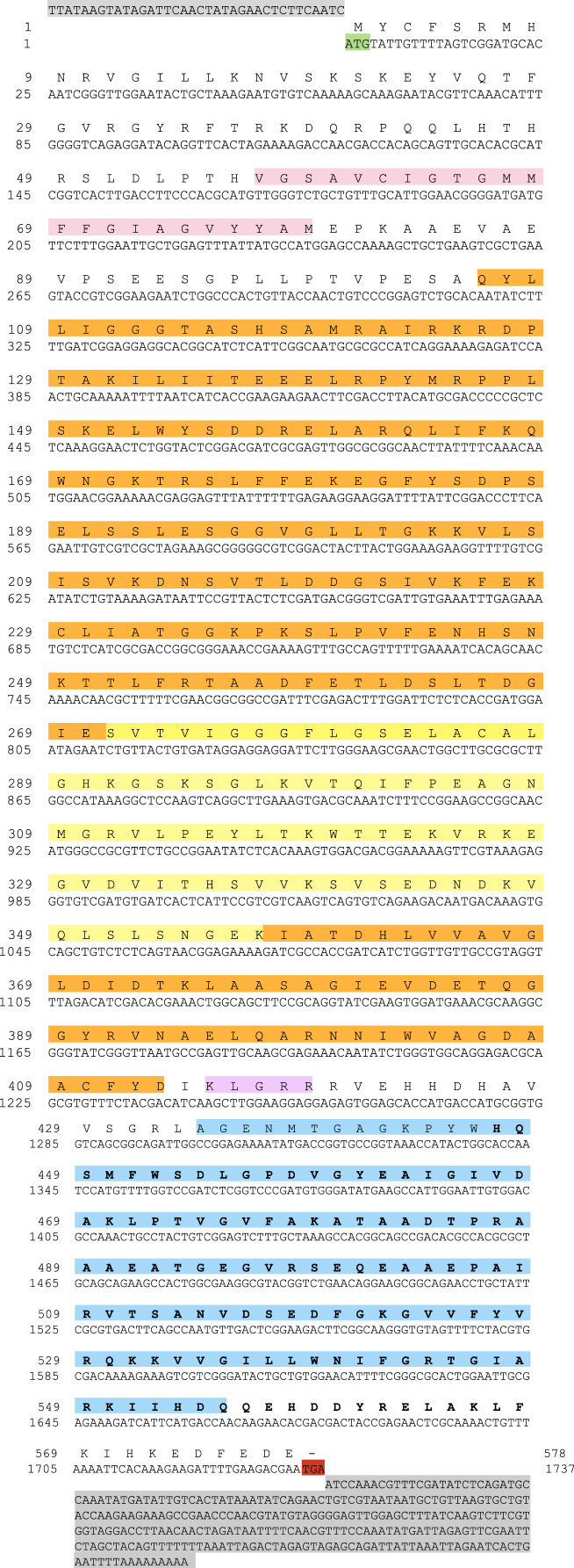
cDNA and deduced amino acid sequence of BsAIF1. START and STOP codon are in green and red, respectively; 5′ and 3′ UTR are in grey. Orange: pyridine nucleotide-disulphide domain (PNDD); yellow: NADH-binding PNDD; light blue: C-terminal domain of dimerisation; light purple: nuclear localisation motif. Bold: the N-terminal transmembrane domain.

**Fig. 5:**
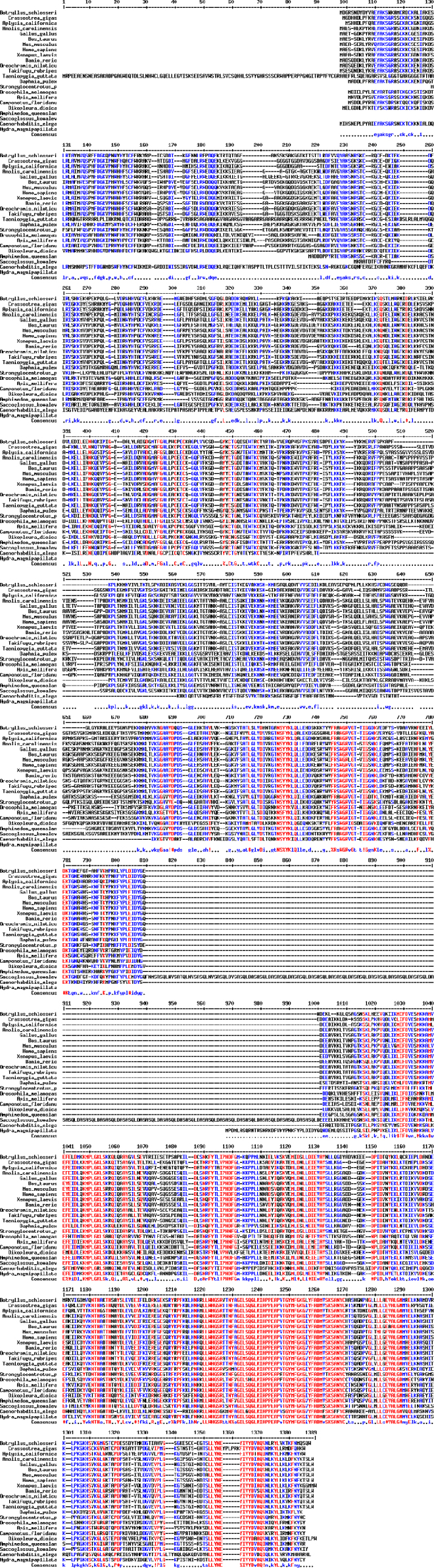
Alignments of the deduced amino acid sequence of BsPARP1 with known orthologous sequences from both vertebrate and invertebrates.

**Fig. 6:**
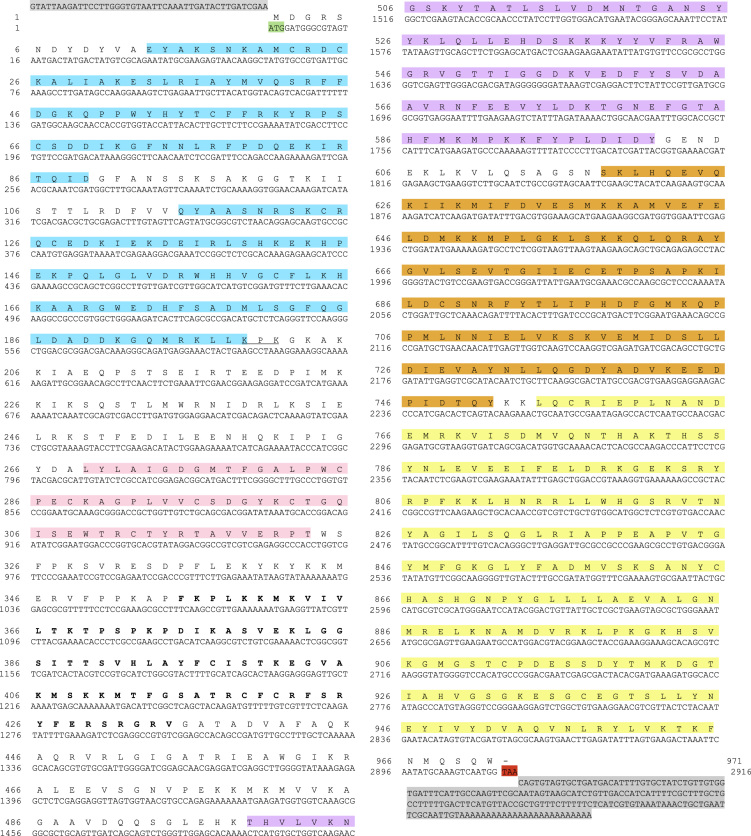
cDNA and deduced amino acid sequence of BsPARP1. START and STOP codon are in green and red, respectively; 5′ and 3′ UTR are in grey. Light blue: N-terminal zinc-finger domains; pink: PADR1 (poly(ADP-ribose)-synthase 1) domain; bold: BRCT (BRCA1 (breast cancer susceptibility protein C-terminus) domain; light purple: WGR (tryptophane-, glycine-, arginine-rich) motif; orange; C-terminal regulatory PARP domain; yellow: C-terminal catalytic PARP domain.

**Fig. 7:**
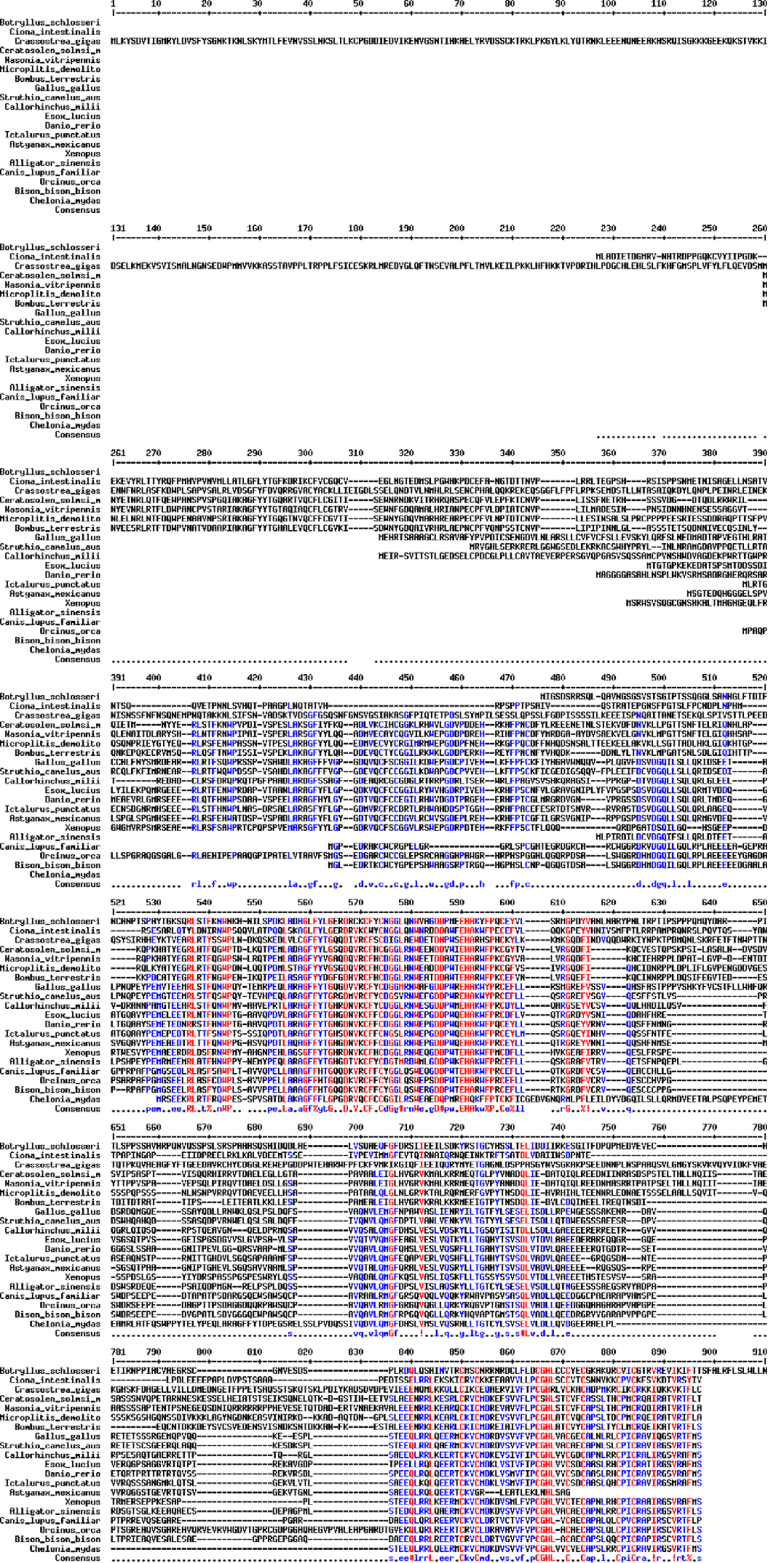
Alignments of the deduced amino acid sequence of BsIAP7 with known orthologous sequences from both vertebrate and invertebrates.

**Fig. 8:**
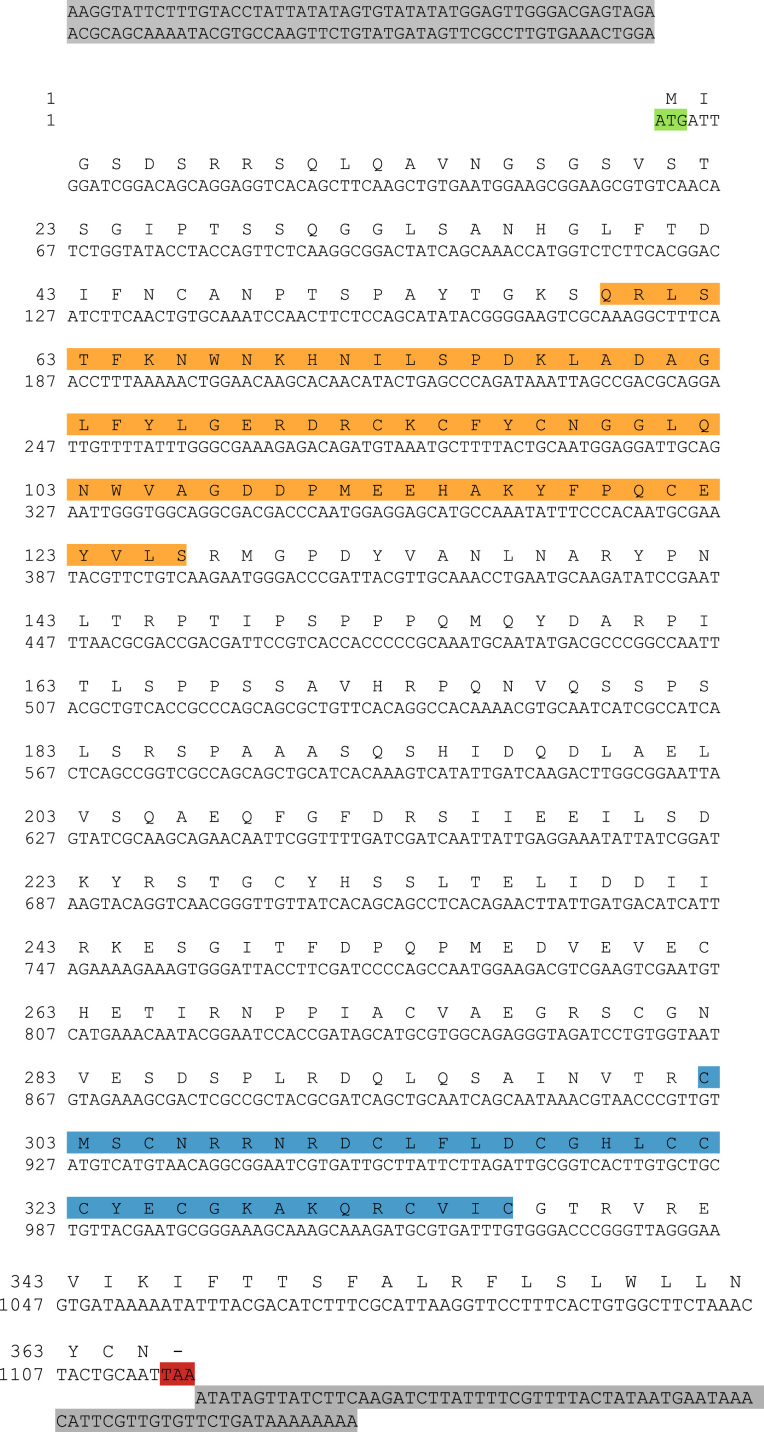
cDNA and deduced amino acid sequence of BsIAP7. START and STOP codon are in green and red, respectively; 5′ and 3′ UTR are in grey. Orange: BIR domain; blue: RING (really interesting new gene) finger domain.

The sequences from GenBank, reported in Supplementary Tables 2–5, were used for alignments and sequence comparisons with the sequences of BsBax, BsAIF1, BsPARP1, BsIAP7, respectively. The latter were deposited in GenBank and corresponding to the accession numbers GenBank: KU948200, GenBank: KU948201, GenBank: KU948202 and GenBank: KU948203, respectively (Fig. 1, Fig. 2, Fig. 3, Fig. 4, Fig. 5, Fig. 6, Fig. 7, Fig. 8).

The predicted amino acid sequences of BsBax, BsAIF1, BsPARP1, BsIAP7 are reported in Figs. 2, 4, 6 and 8, respectively. They were aligned with known orthologous sequences from both vertebrate and invertebrates using the MUSCLE programme [3] and data are reported in Figs. 1, 3, 5, and 7, respectively. Identity analysis of the deduced amino acid sequences were performed using BLAST (http://blast.ncbi.nlm.nih.gov/Blast.cgi) and LALIGN (http://www.ch.embnet.org/software/LALIGN_form.html) [4] and are reported in Supplementary Tables 2–5.

## Footnotes

Appendix B Transparency data associated with this article can be found in the online version at doi:10.1016/j.dib.2016.05.017.
