# Supplementary material for: Data on four apoptosis-related genes in the colonial tunicate Botryllus schlosseri
Source: Data Brief. 2016 May 20;8:142–52. doi: 10.1016/j.dib.2016.05.017 (PMC4889877; doi:10.1016/j.dib.2016.05.017)
Supplement: Supplementary file 1 — Supplementary material [file mmc1.zip › Table1.docx]

| **Primer** | **Sequence** | **Description** | **Amplicon Lenght (bp)** |
| --- | --- | --- | --- |
| (dT)Anchor | GTTTTCCCACGACTTTTTTTTTTTTTTTTTT | cDNA synthesis |  |
| Anchor | GTTTTCCCACGAC | 3’ RACE Anchor |  |
| AAP | GGCCACGCGTCGACTAGTACGGGIIGGGIIGGGIIG | 5’ RACE Anchor |  |
| BAXF | GATCAAGCGAAGAAGGAAGCAGGG | PCR of BsBAX and riboprobe | 725 |
| BAXR | GTTGCGGCAGACAGAGAAACCAC | for ISH |  |
| AIFF | GCGGGGGCGTCGGACTACTTAC | PCR of BsAIF and riboprobE | 928 |
| AIFR | GCCGCTTCCTGTTCAGACCGTAC | for ISH |  |
| PARPF | GATCGGAGCAACGAGGATCGA | PCR of BsPARP and riboprobe | 820 |
| PARPR | TCCAGCAGGCTGTCGATCAT | for ISH |  |
| IAPF | ATTCCGTCACCACCCCCG | PCR of BsIAP and riboprobe | 396 |
| IAPR | GGATCTACCCTCTGCCACGC | for ISH |  |
| BAXF-3’ | CCTTGCGTGTTATTGGAG | 3' RACE of BsBAX | 1526 with Anchor |
| BAXR-5’ | CCTGGTCTGGCGGTTCTCGGTC | 5’ RACE of BsBAX | 249 with AAP |
| AIFF-3’ | GGACGCCAAACTGCCTACTGTCGG | 3' RACE of BsAIF | 649 with Anchor |
| AIFR-5’ | GTACGGTCTGAACAGGAAGCGGC | 5' RACE of BsAIF | 1580 with AAP |
| ParpF-3’ | GGACTGTTATTGCTCGCTGAA | 3’ RACE of BsPARP | 501 with Anchor |
| PARPR-5’ | CAAAGTACAACCCCTTGCC | 5’ RACE of BsPARP | 2628 with AAP |
| PARPR-5’N | GCTCCAACCCAGACTGCT | 5’ RACE of BsPARP Nested | 1551 with AAP |
| IAPF-3’ | GGAAAGCAAAGCAAAGATGC | 3' RACE of BsIAP | 218 with Anchor |
| IAPR-5’ | AAGCTGTGACCTCCTGCTGT | 5' RACE of BsIAP | 170 with AAP |

**Table 1**. PCR primers used in this study.
