# Supplementary material for: Data on four apoptosis-related genes in the colonial tunicate Botryllus schlosseri
Source: Data Brief. 2016 May 20;8:142–52. doi: 10.1016/j.dib.2016.05.017 (PMC4889877; doi:10.1016/j.dib.2016.05.017)
Supplement: Supplementary file 1 — Supplementary material [file mmc1.zip › Table2.docx]

| **Species** | **Accession Number** | **% of identity with BsBAX** |
| --- | --- | --- |
| *Rattus norvegicus)* | GenBank: AAC26327 | 42.3 |
| *Sus scrofa* | GenBank: XP_003127338 | 41.5 |
| *Mus musculus* | GenBank: NP_031553 | 42.3 |
| *Macaca mulatta* | GenBank: NP_001247945 | 40.8 |
| *Oryctolagus cuniculus* | GenBank: XP_008250583 | 39.7 |
| *Homo sapiens* | GenBank: NP_620116 | 40.8 |
| *Homo sapiens* | GenBank: NP_004315 | 41.8 |
| *Taeniopygia guttata* | GenBank: XP_002199710 | 40.4 |
| *Anolis carolinensis* | GenBank: XP_003226241 | 43.6 |
| *Xenopus laevis* | GenBank: NP_001079104 | 43.4 |
| *Xenopus (Silurana) tropicalis* | GenBank: NP_989185 | 42.6 |
| *Onchorynchus mykiss* | GenBank: ACO08752 | 34.3 |
| *Danio rerio* | GenBank: NP_571637 | 41.6 |
| *Ictalurus punctatus* | GenBank: NP_001187866 | 41.4 |
| *Oreochromis niloticus* | GenBank: XP_003456606 | 36.8 |
| *Salmo salar* | GenBank: ACI68449 | 35.5 |
| *Esox lucius* | GenBank: ACO13345 | 35.6 |
| *Ciona intestinalis* | GenBank: XP_002123003 | 38.4 |
| *Strongylocentrotus purpuratus* | GenBank: XP_791118 | 34.7 |
| *Crassostrea gigas* | GenBank: EKC42310 | 37.2 |
| *Drosophila melanogaster* | GenBank: NP_788278 | 17.9 |
| *Hydra magnipapillata* | GenBank: XP_002157460 | 32.1 |
| *Acropora millepora* | GenBank: ABX61041 | 29.9 |

**Table 2**. Percentage of identity between BsBAX and orthologous proteins.
