# Supplementary material for: Data on four apoptosis-related genes in the colonial tunicate Botryllus schlosseri
Source: Data Brief. 2016 May 20;8:142–52. doi: 10.1016/j.dib.2016.05.017 (PMC4889877; doi:10.1016/j.dib.2016.05.017)
Supplement: Supplementary file 1 — Supplementary material [file mmc1.zip › Table3.docx]

| **Species** | **GenBank Accession Number** | **% of identity with BsAIF1** |
| --- | --- | --- |
| *Homo sapiens* | GenBank: NP_004199 | 57.5 |
| *Oryctolagus cuniculus* | GenBank: XP_002720337 | 59.3 |
| *Sus scrofa* | GenBank: NP_001284561 | 56.7 |
| *Rattus norvegicus* | GenBank: NP_112646 | 57.1 |
| *Meleagris gallopavo* | GenBank: XP_003208346 | 60.7 |
| *Taeniopygia guttata* | GenBank: XP_002198740 | 60.7 |
| *Gallus gallus* | GenBank: NP_001007491 | 60.5 |
| *Anolis carolinensis* | GenBank: XP_008118433 | 60 |
| *Xenopus (Silurana) tropicalis* | GenBank: NP_001017244 | 62.4 |
| *Danio rerio* | GenBank: NP_956396 | 61.9 |
| *Takifugu rubripes* | GenBank: XP_003971249 | 61.3 |
| *Oreochromis niloticus* | GenBank: XP_003456194 | 61.7 |
| *Oryzias latipes* | GenBank: XP_004076557 | 61.7 |
| *Tetraodon nigroviridis* | GenBank: CAG02984 | 60.5 |
| *Ciona intestinalis* | GenBank: XP_002131727 | 60.1 |
| *Saccoglossus kovalevski* | GenBank: XP_006825404 | 58.6 |
| *Strongylocentrotus purpuratus* | GenBank: XP_783530 | 62.6 |
| *Crassostrea gigas* | GenBank: EKC36189 | 49.1 |
| *Apis mellifera* | GenBank: XP_006563283 | 51.5 |
| *Tribolium castaneum* | GenBank: XP_972831 | 53.7 |
| *Camponotus floridanus* | GenBank: EFN62354 | 51 |
| *Culex quinquefasciatus* | GenBank: XP_001844711 | 49.7 |
| *Drosophila melanogaster* | GenBank: NP_608649 | 48.1 |
| *Loa loa* | GenBank: EFO16709 | 46.9 |
| *Caenorhabditis elegans* | GenBank: NP_499564 | 39.6 |
| *Hydra magnipapillata* | GenBank: XP_002156723 | 56 |
| *Suberites domuncula* | GenBank: CAL36989 | 47.8 |
| *Amphimedon queenslandica* | GenBank: XP_003385522 | 46.8 |

**Table 3**. Percentage of identity between BsAIF1 and orthologous proteins.
