# Supplementary material for: Data on four apoptosis-related genes in the colonial tunicate Botryllus schlosseri
Source: Data Brief. 2016 May 20;8:142–52. doi: 10.1016/j.dib.2016.05.017 (PMC4889877; doi:10.1016/j.dib.2016.05.017)
Supplement: Supplementary file 1 — Supplementary material [file mmc1.zip › Table4.docx]

| **Species** | **Accession Number** | **% of identity with BsPARP1** |
| --- | --- | --- |
| *Homo sapiens* | GenBank: NP_001609 | 49.1 |
| *Mus musculus* | GenBank: P11103 | 48.6 |
| *Bos taurus* | GenBank: NP_777176 | 48 |
| *Gallus gallus* | GenBank: P26446 | 49.5 |
| *Taeniopygia guttata* | GenBank: XP_002194832 | 48.3 |
| *Anolis carolinensis* | GenBank: XP_003216114 | 48.7 |
| *Xenopus laevis* | GenBank: NP_001081571 | 48.8 |
| *Oreochromis niloticus* | GenBank: XP_003449904 | 50.4 |
| *Takifugu rubripes* | GenBank: XP_003971725 | 49.5 |
| *Danio rerio* | GenBank: NP_001038407 | 49.2 |
| *Ciona intestinalis* | GenBank: XP_009860226 | 55.0 |
| *Oicopleura dioica* | GenBank: CBY18306 | 42.6 |
| *Saccoglossus kovalevski* | GenBank: XP_006812383 | 38.3 |
| *Strongylocentrotus purpuratus* | GenBank: XP_001177436 | 42.9 |
| *Crassostrea gigas* | GenBank: EKC34863 | 46.9 |
| *Aplysia californica* | GenBank: NP_001191521 | 45.5 |
| *Camponotus floridanus* | GenBank: EFN69632 | 43.8 |
| *Daphnia pulex* | GenBank: EFX75651 | 43.4 |
| *Apis mellifera* | GenBank: XP_624477 | 43 |
| *Drosophila melanogaster* | GenBank: NP_001104452 | 39.2 |
| *Caenorhabditis elegans* | GenBank: NP_491072 | 30.5 |
| *Hydra magnipapillata* | GenBank: XP_002163577 | 60.1 |
| *Amphimedon queenslandica* | GenBank: XP_003385071 | 43.8 |

**Table 4**. Percentage of identity between BsPARP1 and orthologous proteins.
