# Supplementary material for: Data on four apoptosis-related genes in the colonial tunicate Botryllus schlosseri
Source: Data Brief. 2016 May 20;8:142–52. doi: 10.1016/j.dib.2016.05.017 (PMC4889877; doi:10.1016/j.dib.2016.05.017)
Supplement: Supplementary file 1 — Supplementary material [file mmc1.zip › Table5.docx]

| **Species** | **Accession Number** | **% of identity with BsIAP7** |
| --- | --- | --- |
| *Canis lupus familiaris* | GenBank: XP_543094.1 | 31.7 |
| *Orcinus orca* | GenBank: XP_004282448.1 | 28.7 |
| *Bison bison bison* | GenBank: XP_010834953.1 | 26.8 |
| *Gallus gallus* | GenBank: XP_417413.4 | 30.0 |
| *Struthio camelus australis* | GenBank: XP_009669159.1 | 30.7 |
| *Alligator sinensis* | GenBank: XP_006021704.1 | 29.9 |
| *Chelonia mydas* | GenBank: EMP35847.1 | 27.3 |
| *Xenopus laevis* | GenBank: NP_001082290.1 | 30.7 |
| *Astyanax_mexicanus* | GenBank: XP_007229030.1 | 28.7 |
| *Ictalurus punctatus* | GenBank: AFL70282.1 | 29.0 |
| *Callorhinchus milii* | GenBank: XP_007887106.1 | 29.0 |
| *Danio rerio* | GenBank: XP_005162039.1 | 31.3 |
| *Esox lucius* | GenBank: XP_010874145.1 | 28.1 |
| *Ciona intestinalis* | GenBank: XP_002125780.2 | 30.7 |
| *Crassostrea gigas* | GenBank: EKC32616.1 | 27.6 |
| *Ceratosolen solmsi marchali* | GenBank: XP_011494114.1 | 26.4 |
| *Bombus terrestris* | GenBank: XP_003393059.1 | 23.4 |
| *Nasonia vitripennis* | GenBank: XP_001606042.2 | 26.7 |
| *Microplitis demolitor* | GenBank: XP_008554578.1 | 24.7 |

**Table 5**. Percentage of identity between BsIAP7 and orthologous proteins.
